# Identification of prognostic alternative splicing signature in gastric cancer

**DOI:** 10.1186/s13690-022-00894-3

**Published:** 2022-05-25

**Authors:** Zhiwu Wang, Qiong Wu, Yankun Liu, Qingke Li, Jingwu Li

**Affiliations:** 1 Department of Chemoradiotherapy, Tangshan People`S Hospital, Tangshan, China; 2 The Cancer Institute, Tangshan People’s Hospital, Tangshan, 063001 China

**Keywords:** Gastric carcinoma, Alternative splicing, Splicing factor, Prognosis

## Abstract

**Background:**

Aberrant alternative splicing (AS) events could be viewed as prognostic indicators in a large number of malignancies. This study aims to identify prognostic AS events, illuminate the function of the splicing variants biomarkers and provide reliable evidence for formulating public health strategies for gastric cancer (GC) surveillance.

**Methods:**

RNA-Seq data, clinical information and percent spliced in (PSI) values were available in The cancer genome atlas (TCGA) and TCGA SpliceSeq data portal. A three-step regression method was conducted to identify prognostic AS events and construct multi-AS-based signatures. The associations between prognostic AS events and splicing factors were also investigated.

**Results:**

We identified a total of 1,318 survival-related AS events in GC, parent genes of which were implicated in numerous oncogenic pathways. The final prognostic signatures stratified by seven types of AS events or not stratified performed well in risk prediction for GC patients. Moreover, five signatures based on AA, AD, AT, ES and RI events function as independent prognostic indicators after multivariate adjustment of other clinical variables. Splicing network also showed marked correlation between the expression of splicing factors and PSI value of AS events in GC patients.

**Conclusion:**

Our findings provide a landscape of AS events and regulatory network in GC, indicating that AS events might serve as prognostic biomarkers and therapeutic targets for GC.

**Supplementary Information:**

The online version contains supplementary material available at 10.1186/s13690-022-00894-3.

## Background

Gastric cancer (GC) remains the public health burden worldwide with high morbidity and mortality, and there are estimated 27,600 new diagnosed cases and 11,010 GC-related deaths in 2018 [1]. A majority of patients were diagnosed at an advanced stage, missing the optimal opportunity of surgical resection, and thus had the dismal prognosis with the overall 5 year survival rate of 32.0% [1]. Therefore, prevention and early diagnosis are the most important pubulic health strategies. Notably, high-throughput omics at DNA, RNA and protein provide effective solutions for screening high-risk individuals with GC. Owing to the combined algorithm based on transcriptomics and genomics, alternative splicing (AS) events as prognostic factors have attracted increasing attention in recent years.

Alternative splicing (AS) is a post-transcriptional regulatory mechanism by which differential splicing of exons occurs, resulting in the diversity of mRNAs and proteins. Over 95% of human genes undergo AS and encode splicing variants with different or even opposite functions [2]. Differential splicing of the same pre-mRNA generates mature isoforms and proteins with different structures and functions. Under normal circumstances, AS is essential for complex biological behaviors. Once disordered, however, abnormal protein isoforms with inserted, missing or altered function domains may drive or promote oncogenic processes. Moreover, it has been widely acknowledged that AS events are closely associated with gastric carcinogenesis and progression [3, 4].

Tumor development is a multi-step, multi-factor processes. Thus, constructing a risk prediction model consisting of diverse biomarkers is an effective and reliable strategy compared to using a single clinicopathological indicator. To improve the predictive accuracy for malignancies such as GC, numerous studies had built diverse prognostic models using several mRNAs, miRNAs and long non-coding RNAs based on transcriptome-wide profiles [5–7]. Although the mechanism of AS events is complicated and remains poorly understood to some extent, their prognostic role has been emphasized in a large number of cancers [8–12]. Considering the role of AS in GC, there is an urgent need to build the prediction model of AS events and screen high-risk patients with GC.

## Methods

### Data curation and preprocessing

The GC cohort, including RNA-Seq (level 3) and corresponding clinical data were downloaded and integrated via R “TCGAbiolinks” package from TCGA data portal (https://portal.gdc.cancer.gov/). The SpliceSeq data for GC was obtained from TCGA SpliceSeq database (https://bioinformatics.mdanderson.org/TCGASpliceSeq). The Percent Spliced In (PSI) value, ranging from 0 to 1, was used in quantifying 7 types of alternative splicing events: Alternate Acceptor site (AA), Alternate Donor site (AD), Alternate Promoter (AP), Alternate Terminator (AT), Exon Skip (ES), Mutually Exclusive Exons (ME) and Retained Intron (RI). For generation of a reliable prognostic model, the included AS events meets the following criterion: (1) more than 75% patients own PSI value; (2) mean PSI of AS event in all samples > 0.1; (3) PSI standard deviation (SD) ≥ 0.05. In addition, patients followed up for over 30 days were enrolled in our study.

### Prognostic model construction via three-step regression analysis

The univariate Cox regression analysis was carried out to screen overall survival (OS)-related AS events. To avoid confounding factors, only patients with follow-up time more than 30 days were enrolled in our study. Upset plot was generated to quantify specific overlapping among seven types of AS events with the Upset package in R. Then, stratified by the splicing type, the top 20 significant (If available) AS events screened above were further selected through LASSO regression (R glmnet) followed by multivariate Cox regression (R survival). Finally, the Cox proportional hazard regression model for each splicing type was constructed. The risk score of each sample was calculated by the following formula: Risk Score = PSI of gene1 × β1 + PSI of gene2 × β2 + ······· + PSI of gene n × βn. Using the median risk score as the cutoff, GC patients were divided into low- and high-risk groups. Kaplan–Meier survival analysis with log rank test was performed to demonstrate the variation between these two groups. In addition, the predictive power of each AS signature was evaluated by calculating the area under the curve (AUC) of the Receiver operating characteristic (ROC).

### GO, KEGG and PPI network

Moreover, parent genes of these AS events were sent for Gene Ontology (GO) and Kyoto Encyclopedia of Genes and Genomes (KEGG) enrichment analyses via “clusterProfiler” package in R software. Then, the significantly enriched GO terms and KEGG pathways (*P* < 0.05) were visualized via barplots. Additionally, these parent genes were submitted to STRING to identify the protein–protein interactions (PPI) which were visualized by the cytoscape software. The key modules and genes were selected in PPI network via using the Cytohubba tool.

### Construction of splicing regulatory network

A total of 404 human genes encoding Splicing factor (SF) were retrieved from the Supplementary files of Seiler M`s paper [13], and their corresponding expression profiles were extracted from the integrated RNA-Seq data of GC. Then, Spearman test was conducted to analyze the correlation between the expression of SF genes and PSI values of survival-associated AS events (*P* < 0.001, coefficient > 0.6), followed by network plotting using the cytoscape software.

## Results

### Overview of AS events and related genes in GC cohort

**Fig.1:**
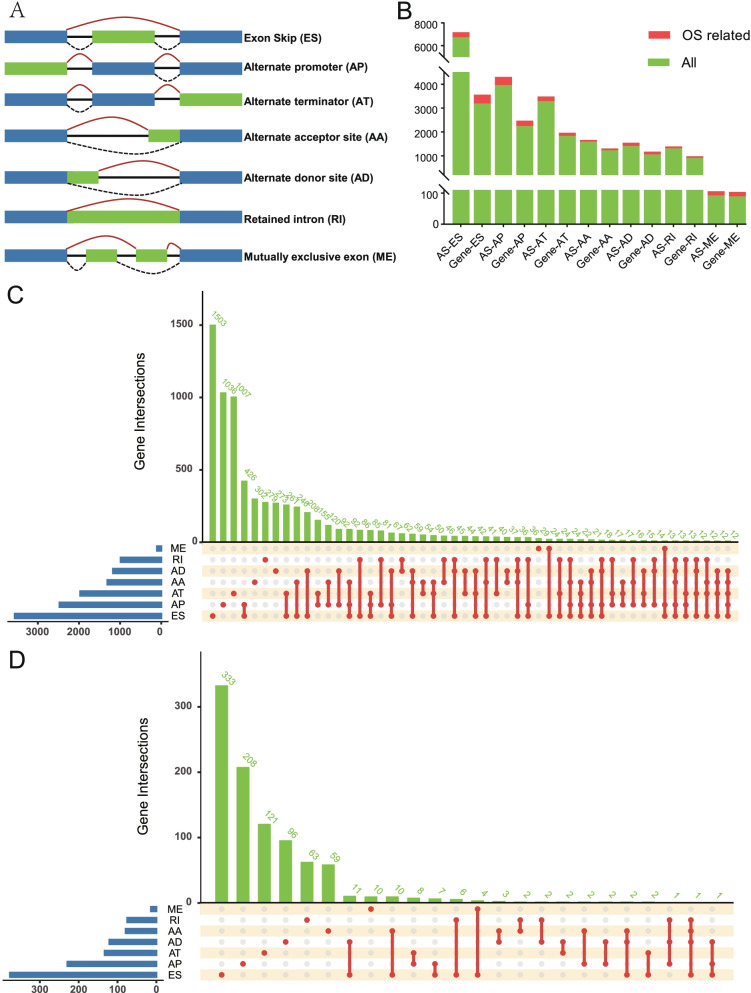
Overview of AS profiles in GC cohort. **A**, Depiction for 7 splicing pattens, including Exon Skip (ES), Alternate Promoter (AP), Alternate Terminator (AT), Alternate Donor Site (AD), Alternate Acceptor Site (AA), Mutually Exclusive Exon (ME) and Retained Intron (RI). **B**, Number of AS events and related genes. The green bars represent all AS events and parent genes, the red bars represent survival related AS events and parent genes. **C**, Upset plot of interactions within 7 types of all AS events. **D**, Upset plot of interactions within 7 types of prognosis-related AS events

AS events could be divided into seven types as illustrated in Fig. 1A, including AA, AD, AP, AT, ES, ME and RI. Through strict filtering, 19,698 AS events from 11,579 parent genes were identified in 364 GC patients, including 7,189 ESs in 3,562 genes, 4,310 APs in 2,474 genes, 3,487 ATs in 1,979 genes, 1,664 AAs in 1,310 genes, 1,542 ADs in 1,174 genes, 1,391 RIs in 985 genes and 106 MEs in 104 genes (Fig. 1B). In addition, the Upset plot indicated that one gene possesses several types of AS events (Fig. 1C).

### Identification of survival-related AS events in GC cohort

Through univariate Cox regression analysis, a total of 1,318 AS events from 957 parent genes were viewed as prognostic ones, including 464 ESs in 377 genes, 352 APs in 229 genes, 200 ATs in 133 genes, 79 AAs in 79 genes, 128 ADs in 121 genes, 81 RIs in 75 genes and 14 MEs in 14 genes (Fig. 1B). Moreover, intersections among these seven types of AS events were exhibited with the Upset plot (Fig. 1D), demonstrating that one gene could hold up to several types of prognostic AS events.

### Bioinformatics analysis of survival associated AS events

**Fig.2:**
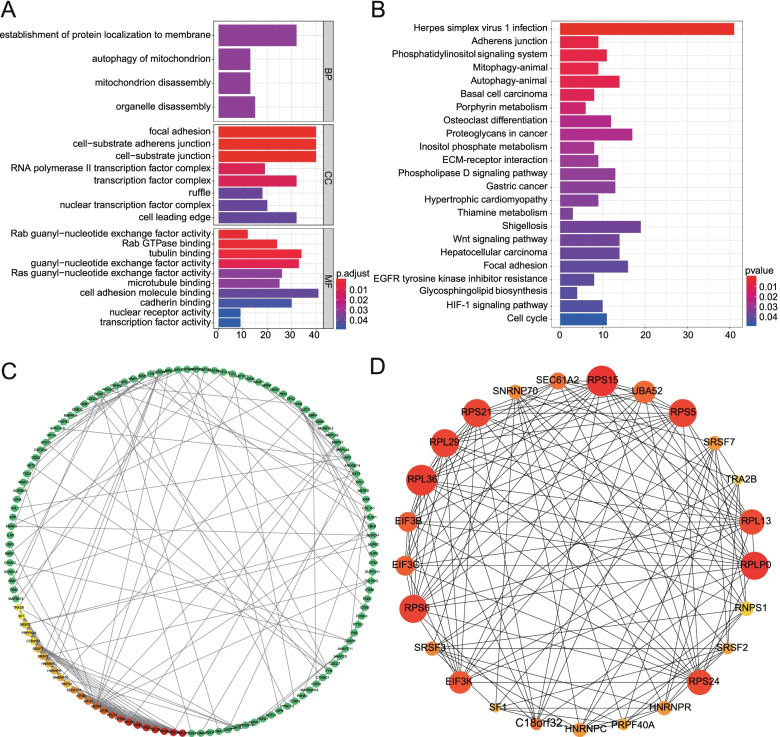
Enrichment analyses on parent genes from Overall survival (OS)-related AS events and their interacting network. **A**, Top 10 iterms (If available) of GO in Biological process (BP), Cellular component (CC) and Molecular function (MF). **B**, Top 30 pathways in KEGG. **C**, Protein Protein Interaction (PPI) network on parent genes of OS-related AS events. **D**, The core module derived from the PPI network. The node size represents the connectivity to other agents. The larger the node in size, the more important the node

**Fig.3:**
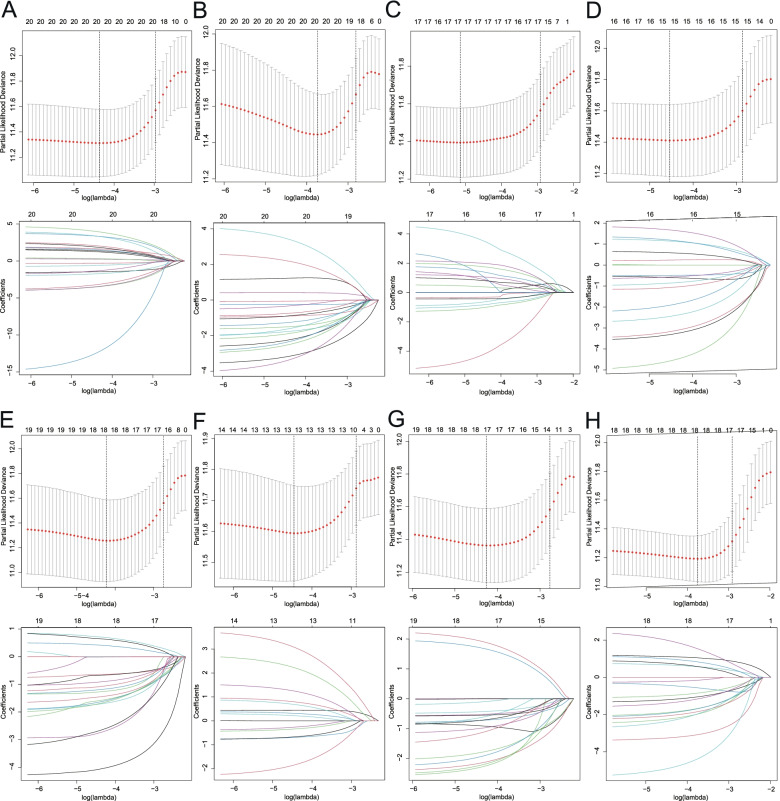
Lasso regression analysis of OS-related AS events by AA, AD, AP, AT, ES, ME, RI type (**A**-**G**) and all types (**H**). Upper, selection of tuning parameter (lambda) in Cox penalized regression analysis via tenfold cross validation. The vertical dotted line on the left and right represents the “lambda.min” and “lambda.lse” criteria, respectively. Lower, dynamic lasso coefficient profiling by Cox-penalized model

To elucidate the potential interference of OS-associated AS events and corresponding proteins, 957 parent genes of 1,391 AS events were sent for bioinformatics analyses, including GO, KEGG and PPI. As a result, 4 terms in biological process, 8 terms in cellular component and 10 terms in molecular function were highlighted via GO analysis (Fig. 2A). Moreover, 11 of 23 remarkably enriched KEGG pathways seem to be implicated in oncogenic processes, including Basal cell carcinoma, Autophagy, Proteoglycans in cancer, ECM-receptor interaction, Gastric cancer, Hepatocellular carcinoma, Focal adhesion, EGFR tyrosine kinase inhibitor resistance, Cell cycle, HIF-1 signaling pathway and Wnt signaling pathway (Fig. 2B). To further explore the significances of these parent genes, a PPI network was constructed which incorporated 373 nodes and 960 edges (Fig. 3C). Moreover, the key module, composed of 25 nodes and 297edges, was processed via CytoHubba tool (Fig. 3D). The parent genes/proteins in the key module were mainly comprised of ribosomal proteins (RPS5, RPS6, RPLP0, etc.) and ribonucleoproteins (HNRNPC, HNRNPR, SNRNP70, etc.).

### Construction of the prognostic signature using AS events for GC patients

**Fig.4:**
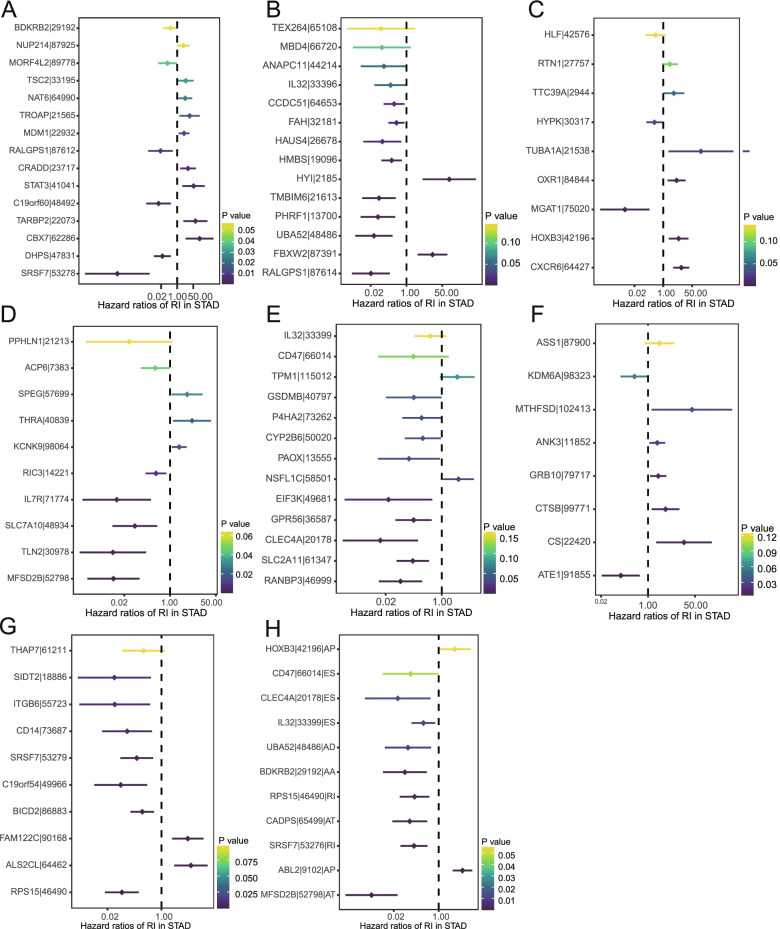
Forest plots of OS-related AS events via Multivariate Cox regression according to stratified (**A-G**: AA, AD, AP, AT, ES, ME, RI) or non-stratified (**H**) strategy. Hazard ratios and 95% Confidence intervals of OS-related AS events

To minimize the counts of the prognostic model, lasso and multivariate Cox regression methods were performed. After lasso regression filtering, 20 varibles (If available) in each splicing type dropped to 20 in AA, 19 in AD, 17 in AP, 16 in AT, 17 in ES, 13 in ME, 15 in RI and 17 in all types, respectively (Fig. 3). Then, the selected AS events were further screened by the multivariate Cox regression, and thus final prognostic models were constructed, containing 15 AA, 14 AD, 9 AP, 10 AT, 13 ES, 8 ME, 10 RI and 11 all AS events (Fig. 4).

**Fig.5:**
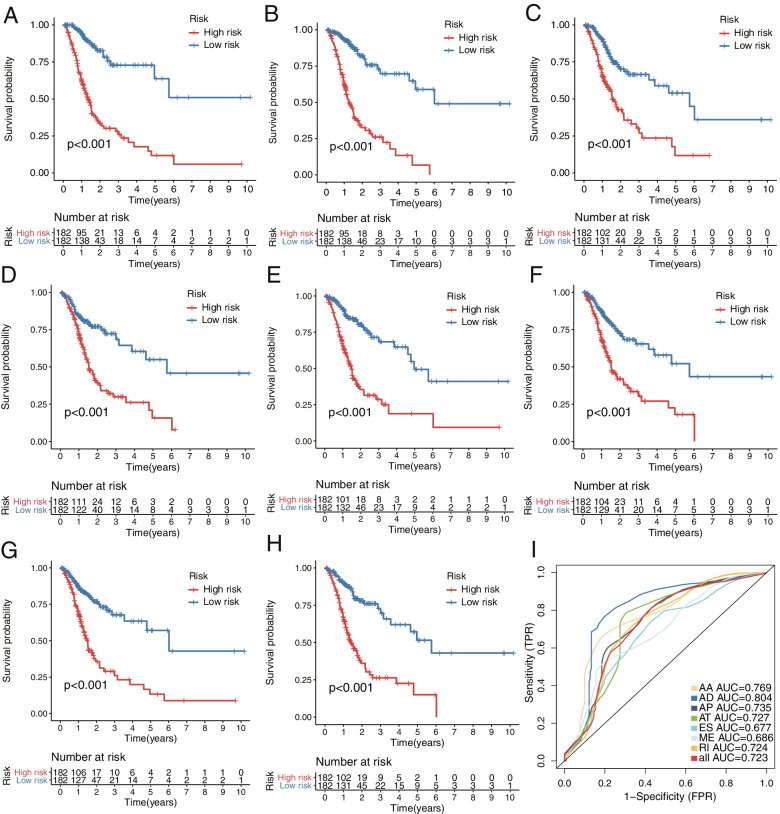
Kaplan–Meier and ROC curves of prediction models in GC. **A-H**, Kaplan–Meier curves depicting the survival probability between high (red)- and low (blue)-riskscore groups calculated by AA, AD, AP, AT, ES, ME, RI type and all types. **I**, ROC curve for AA, AA, AD, AP, AT, ES, ME, RI type and all types

Using the median value of the riskscore as the cutoff, GC patients were classified into high- and low- risk groups. The Kaplan–Meier curves were employed to demonstrate the survival variation of patients between these two groups. Each AS-based prognostic model, stratified or as a whole, indicated the predictive power that patients in the high-risk group had poorer OS than those in the low-risk group (Fig. 5A-H).

**Fig.6:**
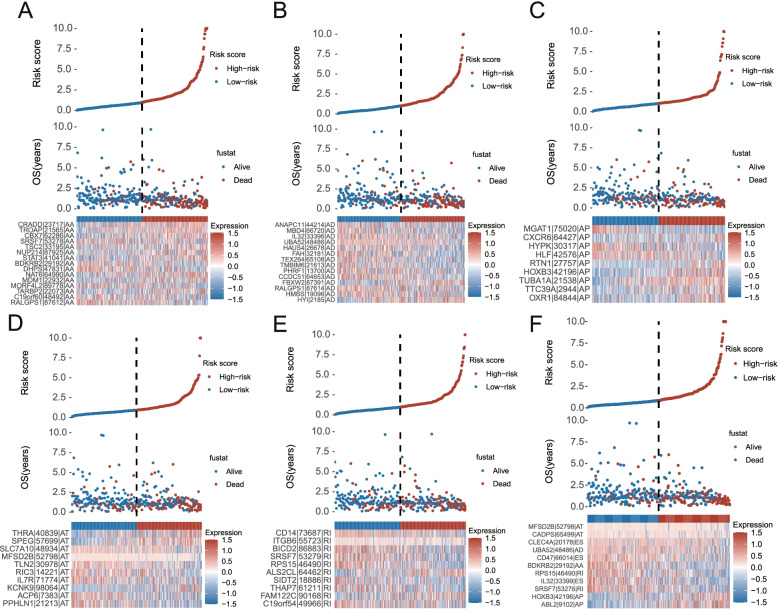
Distributions of survival status (Up), risk score (Middle) and expression profile (Down) of the most reliable prognostic signatures with AUC > 0.7. **A**, AA type; (**B**), AD type; (**C**), AP type; (**D**), AT type; (**E**), RI type; (**F**), non-stratified by the splicing type

ROC curves were generated to assess the predictive accuracy of the eight AS prognostic models. As illustrated in Fig. 5I, the risk score of AD model showed the greatest predictive power with an AUC of 0.804, followed by AA, AP, AT, RI and the model not stratified by AS subtypes. The performance of these prognostic signatures with AUC > 0.7 were further tested in predicting the survival status. With the increasing risk score calculated by any type of AUC > 0.7, there were more patients dead and less patients living (Fig. 6), respectively.

**Fig.7:**
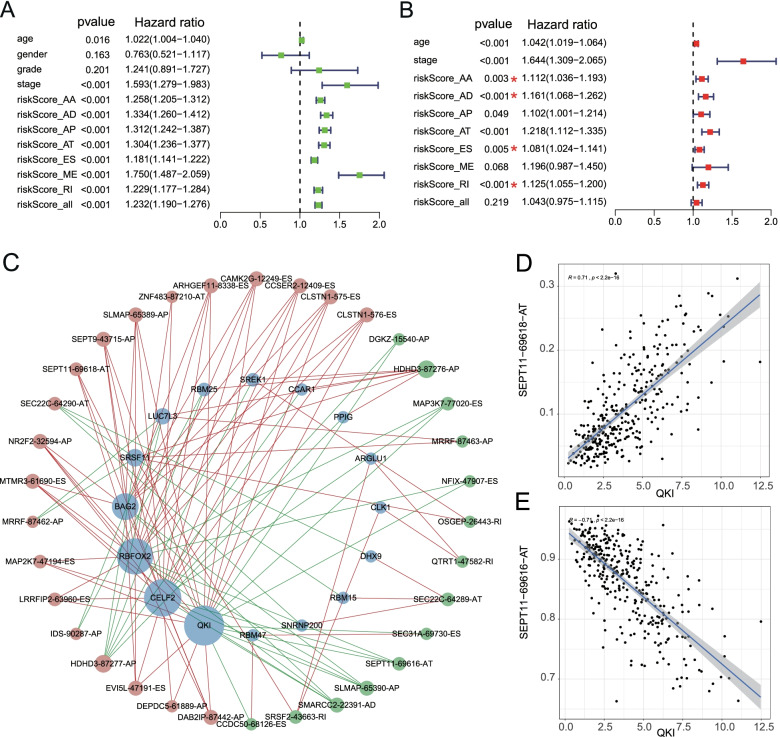
Forest plots of prognostic factors and regulatory network of SF and parent genes. **A-B**, univariate and multivariate Cox regression analyses of AS types combined with age, gender, grade and stage, respectively. Red star indicates independent prognostic AS subtype. **C**, the network of SF expression and PSI value of AS genes. Red, green and blue node represents adverse, favorable AS events and SF, respectively. **D-E**, The correlation plot of SF QKI and PSI value of SEPT11-69,616-AT, SEPT11-69,618-AT, respectively

To determine whether the final model was an independent prognostic factor for GC, AS predictive models along with age, gender, grade and clinical stage, were once again sent for Uni/Multivariate Cox regression analysis. As a result, risk scores calculated by the formula of five AS signatures (AA, AD, AT, ES, RI) were independent prognostic indicators (Fig. 7A, B).

### Interactive analysis of splicing factors and AS events

The regulatory network was built based on the expression of SF genes and PSI values of AS events via using the cytoscape software. As shown in Fig. 7C, prognostic AS events, including 20 risky (red node) and 14 favorable ones (green node), were positively or negatively modulated by the key SF genes (blue node). Remarkably, the same SF could regulate different AS events, and the same AS could be regulated by different SFs. Moreover, a majority of adverse AS events were positively associated with SFs (red line), whereas most favorable AS events were negatively associated with SFs (green line). For the same gene and same splicing type, the SF may play different or even opposite roles producing different isoforms. For example, QKI expression was positively correlated with AT event of SEPT11-69618, but negatively correlated with AT event of SEPT11-69616 (Fig. 7C-E).

## Discussion

Through AS, differential proteins with differential structures and functions can be generated and may be associated with carcinogenesis. Thus, alternative spliced isoforms and AS events could be served as diagnostic, predictive, prognostic biomarkers and even therapeutic targets in a large number of cancers [14–17]. Recent studies demonstrated that Aberrant AS events inducing-variants affected important phenotypes of GC, including proliferation, apoptosis, metastasis and chemotherapy resistance [18–20]. Currently, Chuan Liu et al. [21] and Victoria E S Armero et al. [22] had determined the prediction model of AS events in *Helicobacter pylori*-negative cohort and *Epstein-Barr* virus cohort of GC, respectively. However, the present study depicted the landscape of AS profiles within the entire GC cohort and respectively constructed the risk prediction model stratified by 7 types of AS. In addition, a series of GC-specific and survival associated AS events as well as SFs were identified, which would provide potential intervention targets for GC therapy.

Alternative splicing is one of the critical mechanisms by which the diversity of mRNAs and corresponding proteins occurs in organism. In the present study, a total of 19698 AS events of 11579 genes were detected, showing that AS is a common process in GC and one gene generates several transcripts. Next, we identified 1318 prognosis-related AS events of 957 genes. To explore how these AS events drive tumor initiation, the parent genes were incorporated into GO and KEGG pathway enrichment analyses. These spliced genes were closely associated with HIF-1/Wnt/ErbB/p53 oncogenic pathways. Moreover, we established a PPI network and obtained a key module consisting of 40 hub genes, including splicing factors and ribosomal proteins.

To minimize the number of prognosis-related AS events of the prediction model, we further performed the stepwise lasso and multivariate Cox regressions. Ultimately, seven risk prediction models stratified by AS types as well as one non-stratified prediction model were constructed, respectively. Although prognostic AS signatures of GC had been produced in Zhen Zong et al.`s and Zhang S et al.`s reports [23, 24], our screening strategy seems more reasonable. We first identified OS-related AS events through univariate Cox and lasso regression methods, followed by multivariate Cox regression analysis. Conversely, lasso regression analysis was not employed in Zhen Zong et al.`s report, and multivariate Cox regression methods was not used in Zhang S et al.`s report. The inadequacy of statistical strategy may lead to the overfitting of the risk model and affect the predictive performance of the model.

Importantly, AD showed the most reliable predictive capacity among AS events with the AUC value of 0.804, followed by AA event. Unparalleled with our study, AA event exhibited the highest AUC value, followed by AD event in Zong S et al.`s report. The distinction may arise from the screening strategy of OS-related AS events mentioned above. The AUC > 0.8 is generally considered to be adequate for clinics, so AD event should be highlighted excessively for prospects of clinical application. In addition, via Cox regression analysis with other clinical parameters, AD event was proved to be an independent prognostic indicator of GC. Within the prediction model of AD events, two genes (HYI, FBXW2) were adverse and the other 12 were favorable factors of GC. As a critical E3 ubiquitin ligase, FBXW2 retards tumor proliferation and metastasis via degrading tumor-associated transcription factors or coactivators [25]. However, if FBXW2 is spliced by AD event, it becomes a poor prognostic indicator which may facilitate tumorigenesis and progression of GC.

Mutations or alterations in the expression of regulatory splicing factors could cause aberrant AS events and production of differential spliced variants, thus promoting or inhibiting the oncogenic phenotype in multiple cancers [26, 27]. By constructing the regulatory network between the expression of SFs and PSI value of OS-related AS events, we identified 16 critical SFs, some of which had been documented to exhibit pro-oncogenic or anti-oncogenic behavior in a series of malignancies, including GC. These SFs regulate the expression of the spliced variants derived from the same pre-mRNA via different AS events. Even within the same gene, the same AS event in multiple loci may generate differential spliced variants with the same or opposite behavior. For instance, QKI served as a tumor suppressor and inhibited gastric carcinogenesis via alternative splicing of the histone macroH2A1 [28]. In our bioinformatics analysis, QKI may play a dual role in the development of GC, promoting tumorigenesis via a poor prognostic AS indicator (Fig. 3B) or inhibiting tumorigenesis via a favorable prognostic AS indicator (Fig. 3C). CLK1 had been considered as a novel therapeutic target in GC through phosphoproteomic analysis, and facilitated the proliferation, migration and invasion of GC cells via modulation of the phosphorylation of SRSF2 [29]. The regulatory network in our study also reveals that CLK1 activates the RI event of SRSF2, and generates the spliced variants which favors the prognosis. Interestingly, SRSF2 belongs to the family of SFs itself and contributes to the carcinogenesis of multiple cancers via alternative splicing [30, 31]. Thus, SFs play a pivotal role in tumor development via regulating AS events of key genes.

## Conclusion

The present study constructed an ideal prognostic signature of AS events based on which effective public health strategies could be formulated to monitor high-risk populations with GC.

## Supplementary Information

**Additional file 1: Supplementary Fig.1. **Forest plots of prognosis-related AS events via Univariate Cox regression according to stratified (A-G, AA, AD, AP, AT, ES, ME, RI type) or non-stratified (H) strategy. Hazard ratios and 95% Confidence intervals of top 20 (If available) OS-related AS events.

## Data Availability

The data information of this study was obtained from the Cancer Genome. Atlas (TCGA, https://portal.gdc.cancer.gov/) and the SpliceSeq database (https://bioinformatics.mdanderson.org/TCGASpliceSeq).

## Abbreviations

AS: Aberrant alternative splicing
GC: Gastric cancer
PSI: Percent spliced in
TCGA: The cancer genome atlas
AA: Alternate Acceptor site
AD: Alternate Donor site
AP: Alternate Promoter
AT: Alternate Terminator
ES: Exon Skip
ME: Mutually Exclusive Exons
RI: Retained Intron
OS: Overall survival
AUC: Area under the curve
ROC: Receiver operating characteristic
GO: Gene Ontology
KEGG: Kyoto Encyclopedia of Genes and Genomes
PPI: The protein–protein interaction
SF: Splicing factor

## References

[1] Siegel RL, Miller KD, Jemal A (2020). Cancer statistics, 2020. CA Cancer J Clin.

[2] Chen MX, Zhang KL, Gao B, Yang JF, Tian Y, Das D (2020). Phylogenetic comparison of 5' splice site determination in central spliceosomal proteins of the U1–70K gene family, in response to developmental cues and stress conditions. Plant J.

[3] Liang X, Chen W, Shi H, Gu X, Li Y, Qi Y (2018). PTBP3 contributes to the metastasis of gastric cancer by mediating CAV1 alternative splicing. Cell Death Dis.

[4] Zhu S, Chen Z, Katsha A, Hong J, Belkhiri A, El-Rifai W (2016). Regulation of CD44E by DARPP-32-dependent activation of SRp20 splicing factor in gastric tumorigenesis. Oncogene.

[5] Peng K, Chen E, Li W, Cheng X, Yu Y, Cui Y (2020). A 16-mRNA signature optimizes recurrence-free survival prediction of Stages II and III gastric cancer. J Cell Physiol.

[6] Yang Y, Qu A, Zhao R, Hua M, Zhang X, Dong Z (2018). Genome-wide identification of a novel miRNA-based signature to predict recurrence in patients with gastric cancer. Mol Oncol.

[7] Wang Y, Zhang H, Wang J (2020). Discovery of a novel three-long non-coding RNA signature for predicting the prognosis of patients with gastric cancer. J Gastrointest Oncol.

[8] Zong Z, Li H, Yi C, Ying H, Zhu Z, Wang H (2018). Genome-Wide Profiling of Prognostic Alternative Splicing Signature in Colorectal Cancer. Front Oncol.

[9] Zeng Y, Zhang P, Wang X, Wang K, Zhou M, Long H (2020). Identification of Prognostic Signatures of Alternative Splicing in Glioma. J Mol Neurosci.

[10] Li S, Hu Z, Zhao Y, Huang S, He X (2019). Transcriptome-Wide Analysis Reveals the Landscape of Aberrant Alternative Splicing Events in Liver Cancer. Hepatology.

[11] Zhao D, Zhang C, Jiang M, Wang Y, Liang Y, Wang L (2020). Survival-associated alternative splicing signatures in non-small cell lung cancer. Aging (Albany NY).

[12] Zhou YJ, Zhu GQ, Zhang QW, Zheng KI, Chen JN, Zhang XT (2019). Survival-Associated Alternative Messenger RNA Splicing Signatures in Pancreatic Ductal Adenocarcinoma: A Study Based on RNA-Sequencing Data. DNA Cell Biol.

[13] Seiler M, Peng S, Agrawal AA, Palacino J, Teng T, Zhu P (2018). Somatic Mutational Landscape of Splicing Factor Genes and Their Functional Consequences across 33 Cancer Types. Cell Rep.

[14] Li Y, Sun N, Lu Z, Sun S, Huang J, Chen Z (2017). Prognostic alternative mRNA splicing signature in non-small cell lung cancer. Cancer Lett.

[15] Davy G, Rousselin A, Goardon N, Castéra L, Harter V, Legros A (2017). Detecting splicing patterns in genes involved in hereditary breast and ovarian cancer. Eur J Hum Genet.

[16] Marzese DM, Manughian-Peter AO, Orozco JIJ, Hoon DSB (2018). Alternative splicing and cancer metastasis: prognostic and therapeutic applications. Clin Exp Metastasis.

[17] Martinez-Montiel N, Rosas-Murrieta NH, Anaya Ruiz M, Monjaraz-Guzman E, Martinez-Contreras R (2018). Alternative Splicing as a Target for Cancer Treatment. Int J Mol Sci.

[18] Peng WZ, Liu JX, Li CF, Ma R, Jie JZ (2019). hnRNPK promotes gastric tumorigenesis through regulating CD44E alternative splicing. Cancer Cell Int.

[19] Li Y, Gao X, Wei C, Guo R, Xu H, Bai Z (2020). Modification of Mcl-1 alternative splicing induces apoptosis and suppresses tumor proliferation in gastric cancer. Aging (Albany NY).

[20] Li J, Feng D, Gao C, Zhang Y, Xu J, Wu M (2019). Isoforms S and L of MRPL33 from alternative splicing have isoform‑specific roles in the chemoresponse to epirubicin in gastric cancer cells via the PI3K/AKT signaling pathway. Int J Oncol.

[21] Liu C, Hu C, Li Z, Feng J, Huang J, Yang B (2020). Systematic profiling of alternative splicing in Helicobacter pylori-negative gastric cancer and their clinical significance. Cancer Cell Int.

[22] Armero VES, Tremblay MP, Allaire A, Boudreault S, Martenon-Brodeur C, Duval C (2017). Transcriptome-wide analysis of alternative RNA splicing events in Epstein-Barr virus-associated gastric carcinomas. PLoS One.

[23] Zong Z, Li H, Ning Z, Hu C, Tang F, Zhu X (2020). Integrative bioinformatics analysis of prognostic alternative splicing signatures in gastric cancer. J Gastrointest Oncol.

[24] Zhang S, Hu Z, Lan Y, Long J, Wang Y, Chen X (2020). Prognostic significance of survival-associated alternative splicing events in gastric cancer. Aging (Albany NY).

[25] Yang F, Xu J, Li H, Tan M, Xiong X, Sun Y (2019). FBXW2 suppresses migration and invasion of lung cancer cells via promoting β-catenin ubiquitylation and degradation. Nat Commun.

[26] Dvinge H, Kim E, Abdel-Wahab O, Bradley RK (2016). RNA splicing factors as oncoproteins and tumour suppressors. Nat Rev Cancer.

[27] Bejar R (2016). Splicing Factor Mutations in Cancer. Adv Exp Med Biol.

[28] Li F, Yi P, Pi J, Li L, Hui J, Wang F (2016). QKI5-mediated alternative splicing of the histone variant macroH2A1 regulates gastric carcinogenesis. Oncotarget.

[29] Babu N, Pinto SM, Biswas M, Subbannayya T, Rajappa M, Mohan SV (2020). Phosphoproteomic analysis identifies CLK1 as a novel therapeutic target in gastric cancer. Gastric Cancer.

[30] Luo C, Cheng Y, Liu Y, Chen L, Liu L, Wei N (2017). SRSF2 Regulates Alternative Splicing to Drive Hepatocellular Carcinoma Development. Cancer Res.

[31] Liang Y, Tebaldi T, Rejeski K, Joshi P, Stefani G, Taylor A (2018). SRSF2 mutations drive oncogenesis by activating a global program of aberrant alternative splicing in hematopoietic cells. Leukemia.

