# Supplementary figures and images for: Identification of prognostic alternative splicing signature in gastric cancer
Source: Arch Public Health. 2022 May 25;80:145. doi: 10.1186/s13690-022-00894-3 (PMC9131537; doi:10.1186/s13690-022-00894-3)

**A**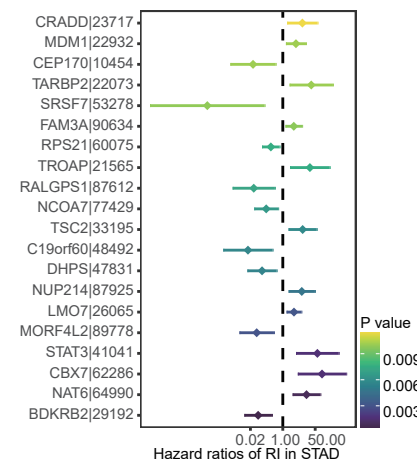**B**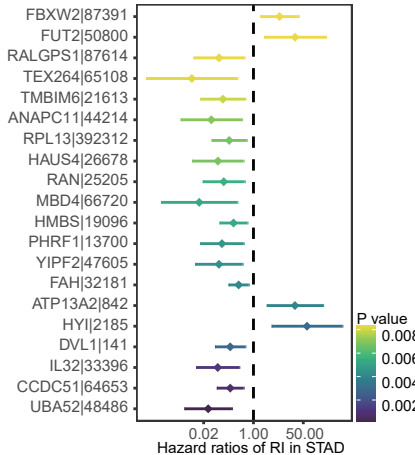**C**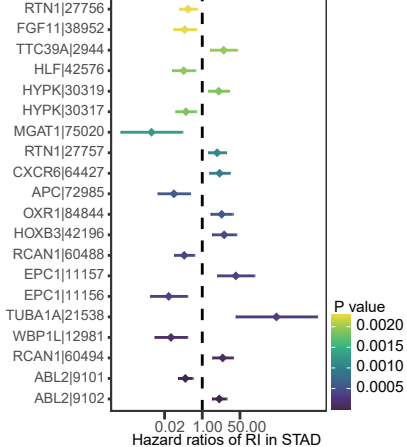**D**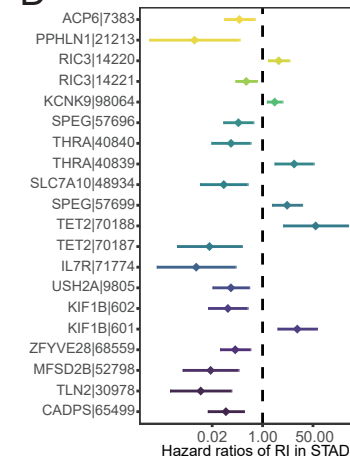**E**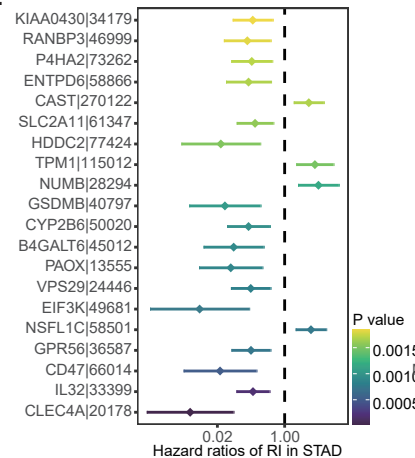**F**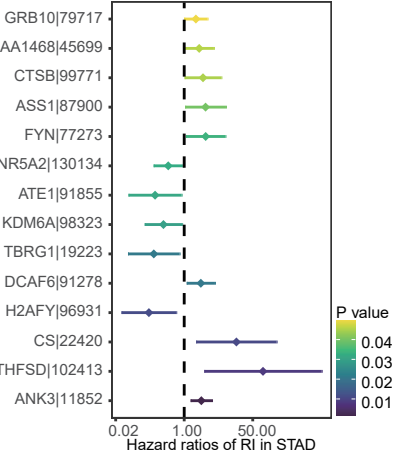**G**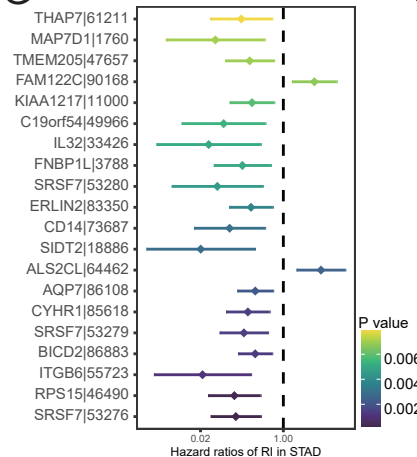**H**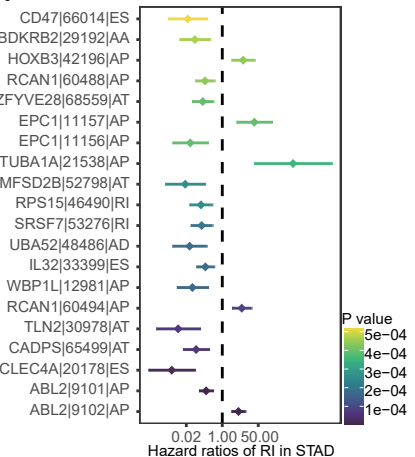

Supplement: Supplementary file 1 — Additional file 1: Supplementary Fig.1. Forest plots of prognosis-related AS events via Univariate Cox regression according to stratified (A-G, AA, AD, AP, AT, ES, ME, RI type) or non-stratified (H) strategy. Hazard ratios and 95% Confidence intervals of top 20 (If available) OS-related AS events. [file 13690_2022_894_MOESM1_ESM.pdf]
